# Supplementary material for: Copy-choice recombination during mitochondrial L-strand synthesis causes DNA deletions
Source: Nat Commun. 2019 Feb 15;10:759. doi: 10.1038/s41467-019-08673-5 (PMC6377680; doi:10.1038/s41467-019-08673-5)
Supplement: Supplementary file 1 — Supplementary Information [file 41467_2019_8673_MOESM1_ESM.docx]

**SUPPLEMENTARY INFORMATION FOR:**

**Copy-choice recombination during mitochondrial L-strand synthesis causes DNA deletions**

*Persson et al.*


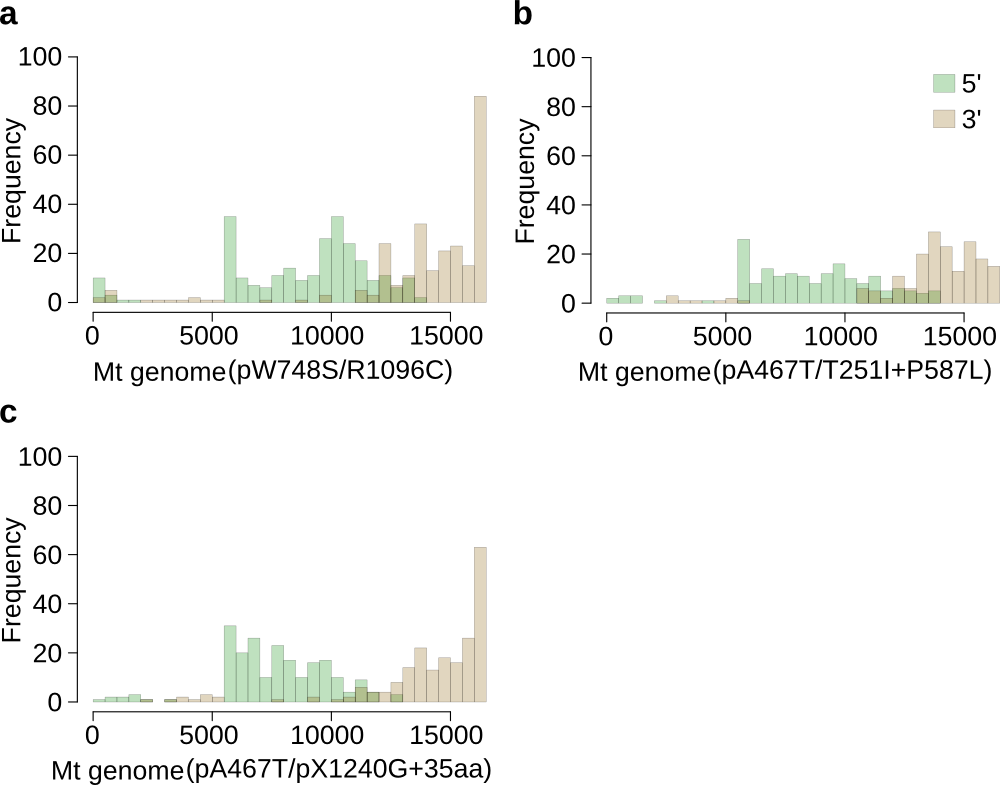


**Supplementary Figure 1. Distribution of 5**′ **and 3**′ **breakpoint positions on the mtDNA**.

Predicted using whole genome sequencing of skeletal muscle DNA from patients with pathogenic, heterozygous *POLG* variants. (a) *POLG* p.W748S/p.R1096C (male, 55 yrs) (b) *POLG* p.A467T/p.T251I+P587L (male, 80 yrs) (c) *POLG* p.A467T/p.X1240G+35aa (male 86 yrs).


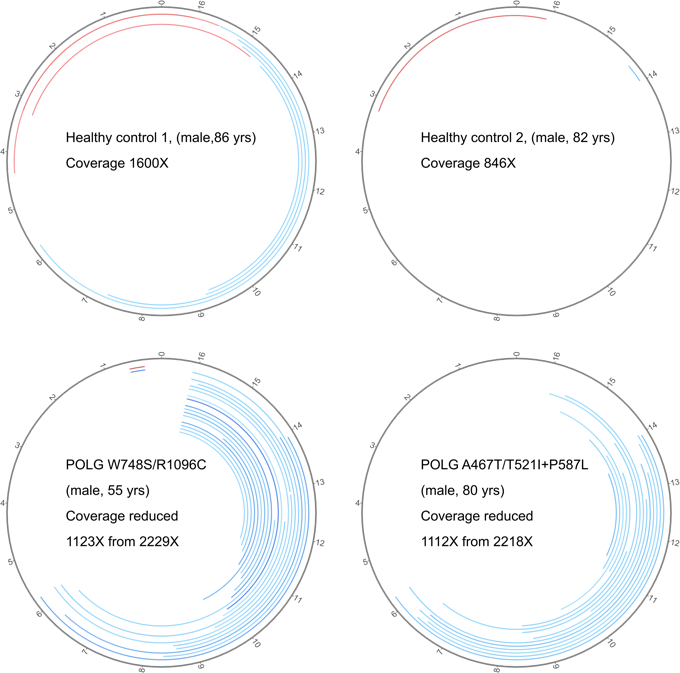


**Supplementary Figure 2. Detected deletions and duplications in *POLG* patient samples after subsampling of reads to reduce coverage**. Sequencing data from two of the *POLG* patient were subsampled to remove 50% of the reads, to compensate for a markedly higher sequencing depth in these samples compared to the healthy controls. This reduced mtDNA coverage to 1123× and 1112× for these two patients, while the third and remaining *POLG* patient sample had a coverage of 1127× without subsampling. The two muscle controls were sequenced to a depth of 846× and 1692×.


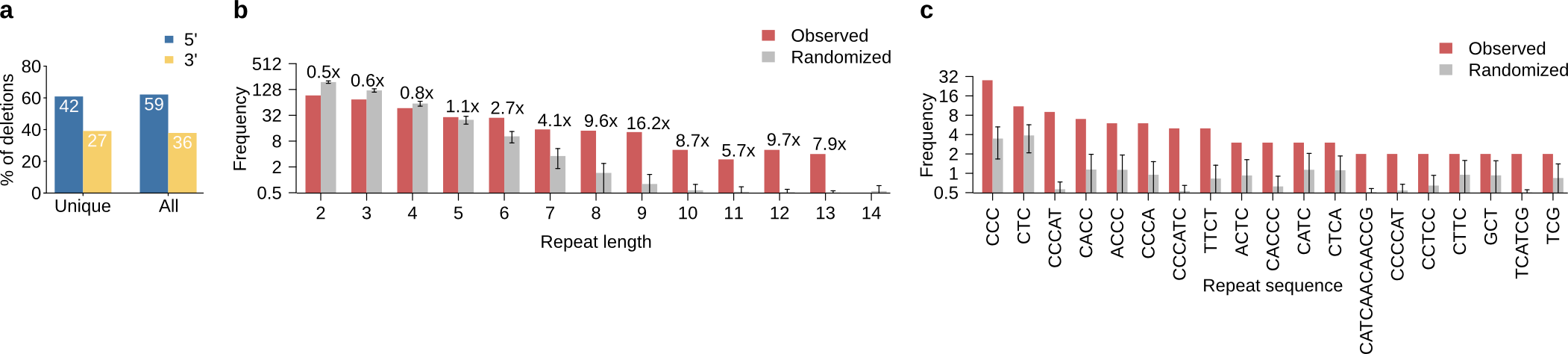


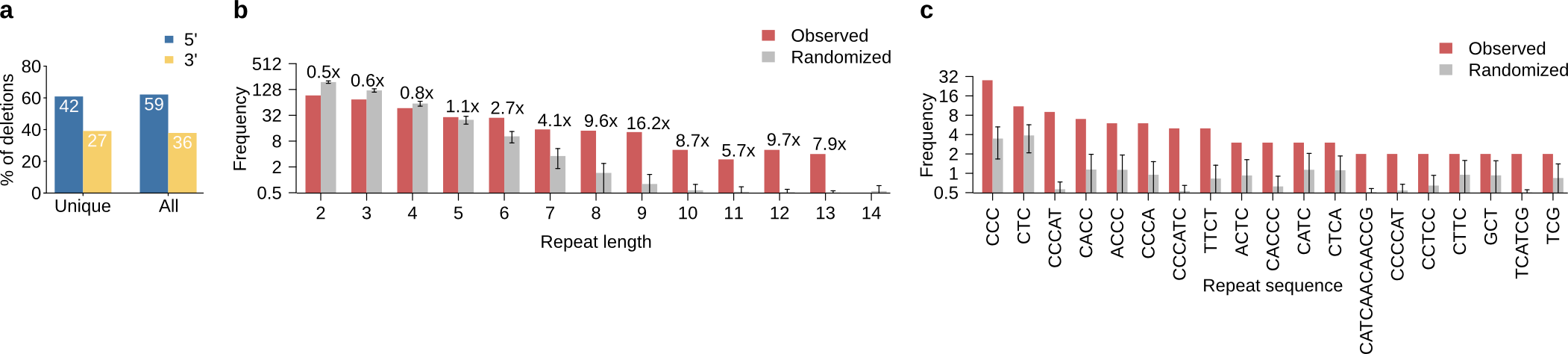


**Supplementary Figure 3. Analysis of mtDNA breakpoints in patient harbouring pathogenic, recessive *TOP3A* variants**.

(a) Analysis of 5′ vs. 3′ retention of imperfect repeats in the mtDNA of a patient (female, 67 yrs) with pathogenic, heterozygous *TOP3A* variant through mitochondrial genome sequencing. Results are shown for unique deletions as well as the complete set of deletions. (b) Frequencies of exact direct repeats overlapping or flanking each pair of breakpoints, considering the longest match for each deletion (red bars). Randomized breakpoints are shown for comparison (grey bars), with error bars indicating the standard deviation (100 randomizations). (c) Frequencies for the most commonly observed repeat patterns. Error bars represent standard deviation for 100 samples of random breakpoints.

**
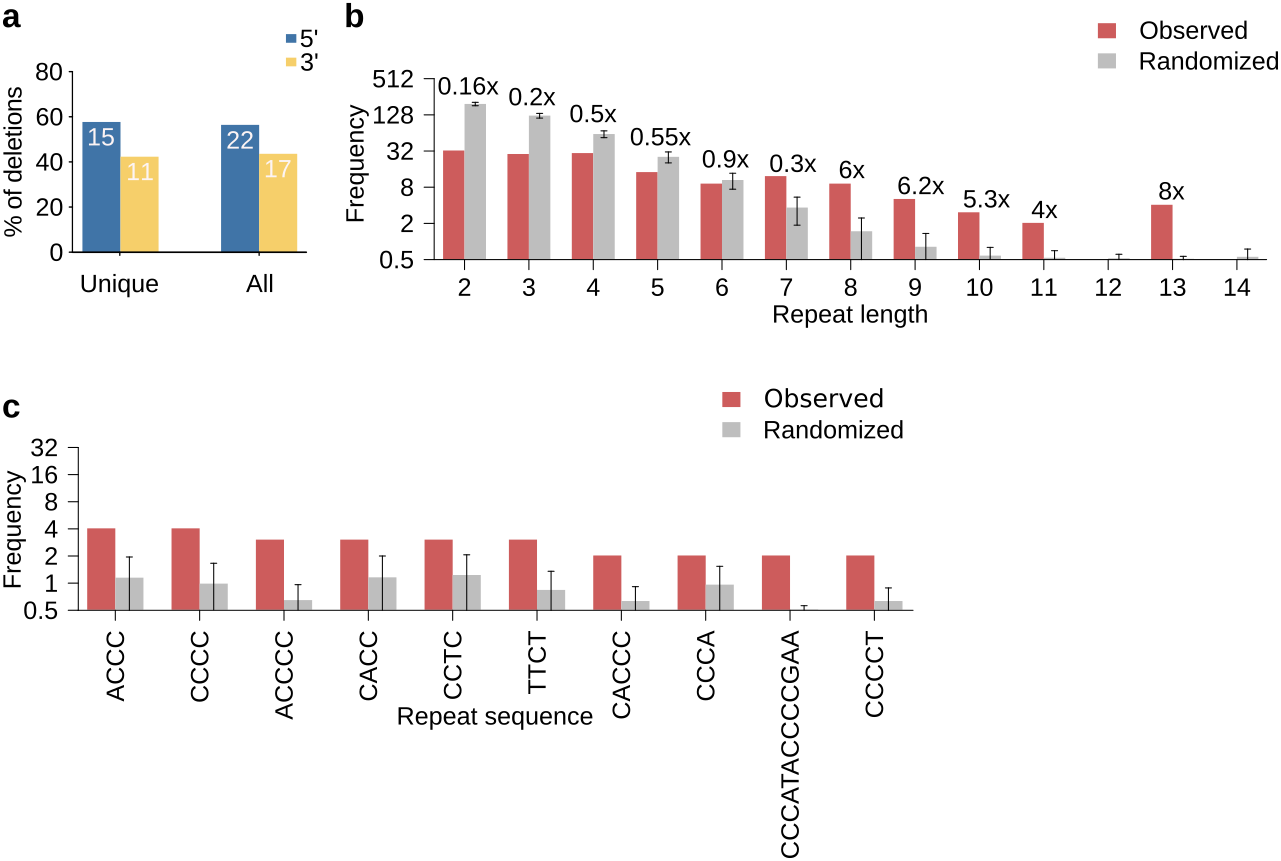
**

**Supplementary Figure 4. Analysis of mtDNA breakpoints in a patient harbouring a pathogenic, dominant *TWNK* variant**.

(a) Analysis of 5′ vs. 3′ retention of imperfect repeats in the mtDNA of a patient (male, 78 yrs) with pathogenic, heterozygous dominant *TWNK* variant through mitochondrial genome sequencing. Results are shown for unique deletions as well as the complete set of deletions. (b) Frequencies of exact direct repeats overlapping or flanking each pair of breakpoints, considering the longest match for each deletion (red bars). Randomized breakpoints are shown for comparison (grey bars), with error bars indicating the standard deviation (100 randomizations). (c) Frequencies for the most commonly observed repeat patterns Error bars represent standard deviation for 100 samples of random breakpoints.

**
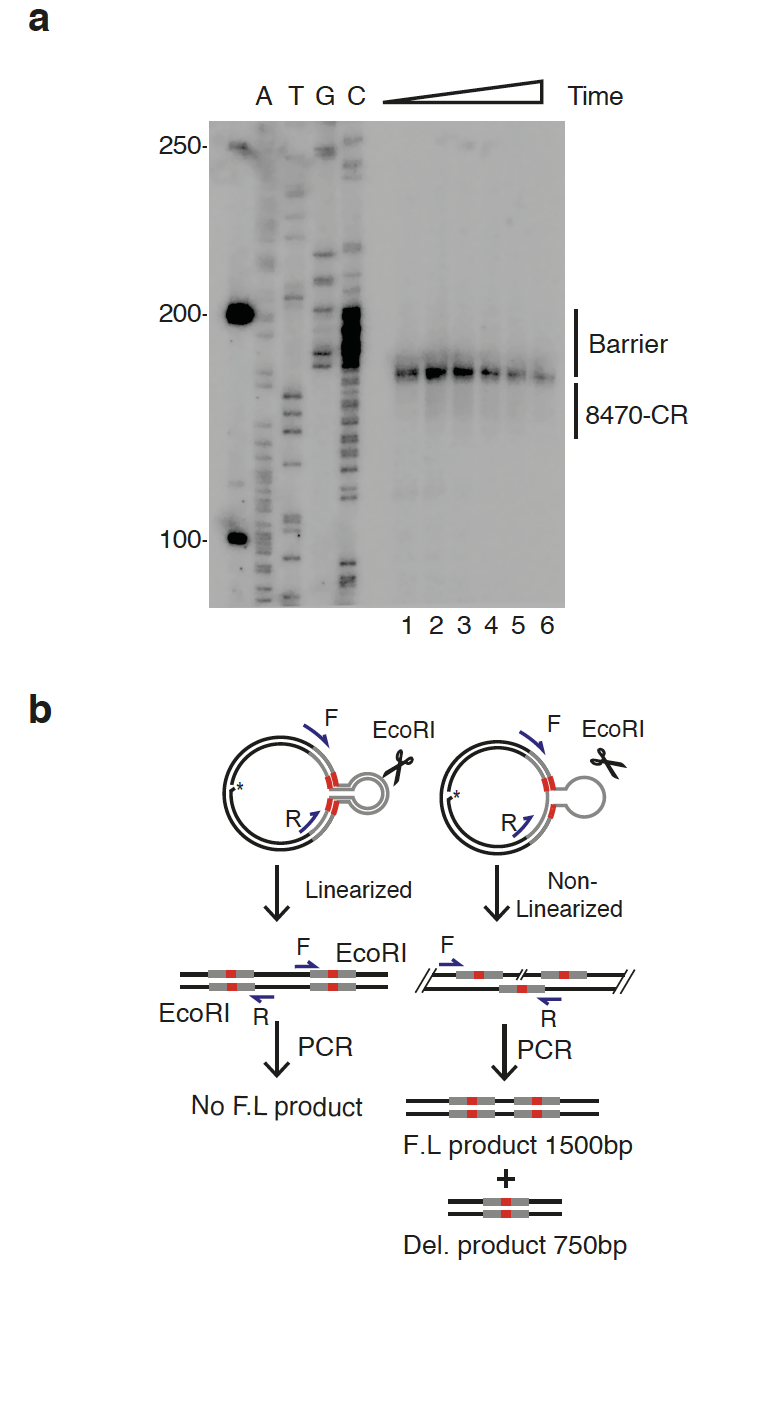
**

**Supplementary Figure 5. Deletion formation during L-strand synthesis.**

(a) To map the site of POLγ stalling observed in Fig. 2c we initiated L-strand DNA synthesis from a primer annealed ~200 bp upstream of 8740-CR. A time course experiment (1, 5, 10, 20, 40, 60 minutes) was performed as described in Methods. The reactions were analysed on 4 % sequencing gel (1 × TBE and 7 M urea) followed by exposure on film. The sequencing ladders were prepared with the same primer using USB Sequenase Version 2.0 (Affymetrix) according to the manufacturer’s protocol. POLγ stalls just before the GC-rich hairpin barrier. (b) Schematic representation of the expected products after EcoRI linearization. EcoRI linearizes the full length (F.L.) dsDNA nicked products (the EcoRI site is indicated in the figure) but does not cleave the heteroduplex formed during copy-choice recombination. Source data are provided as a Source Data file.


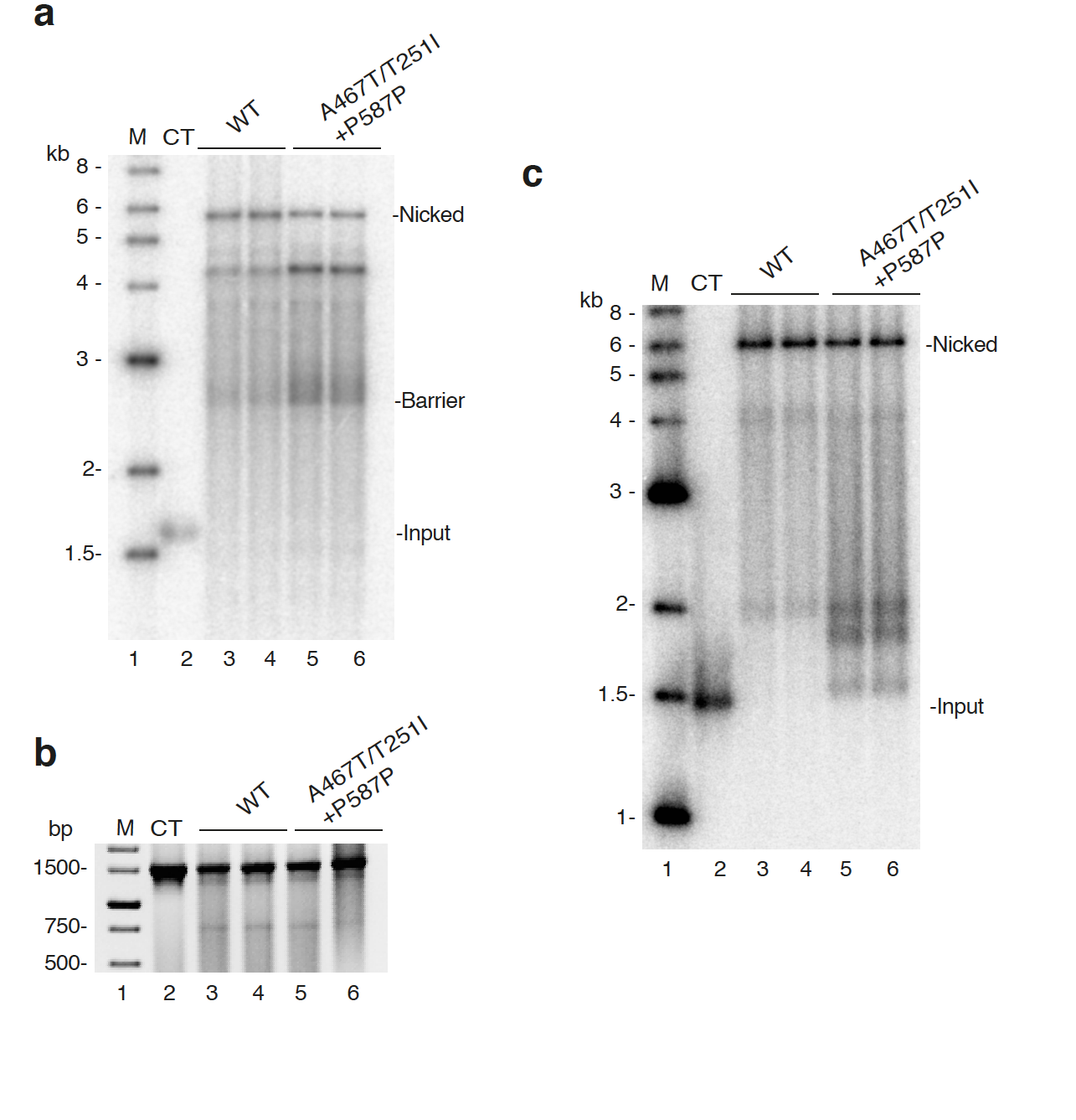
**Supplementary Figure 6. Deletion formation using A467T/T251I+P587L.**

(a) L-strand DNA synthesis using the T1 template. Reactions were performed as described in Methods. Duplicate reactions of wild type or A467T/T251I+P587L mutant POLγ were run in parallel in the presence of 500 μm dNTPs. CT- input template. The reactions were purified by QIAquick PCR purification (QIAGEN) and sent for deep sequencing as described in Methods. (b) The reactions in panel a were analysed by PCR. CT indicates the T1 substrate template without polymerase, which was used as a control. (c) As in panel a but with T5 template and 100 μm dNTPs. The reactions were purified by QIAquick PCR purification (QIAGEN) and sent for deep sequencing as described in Methods. Source data are provided as a Source Data file.

**
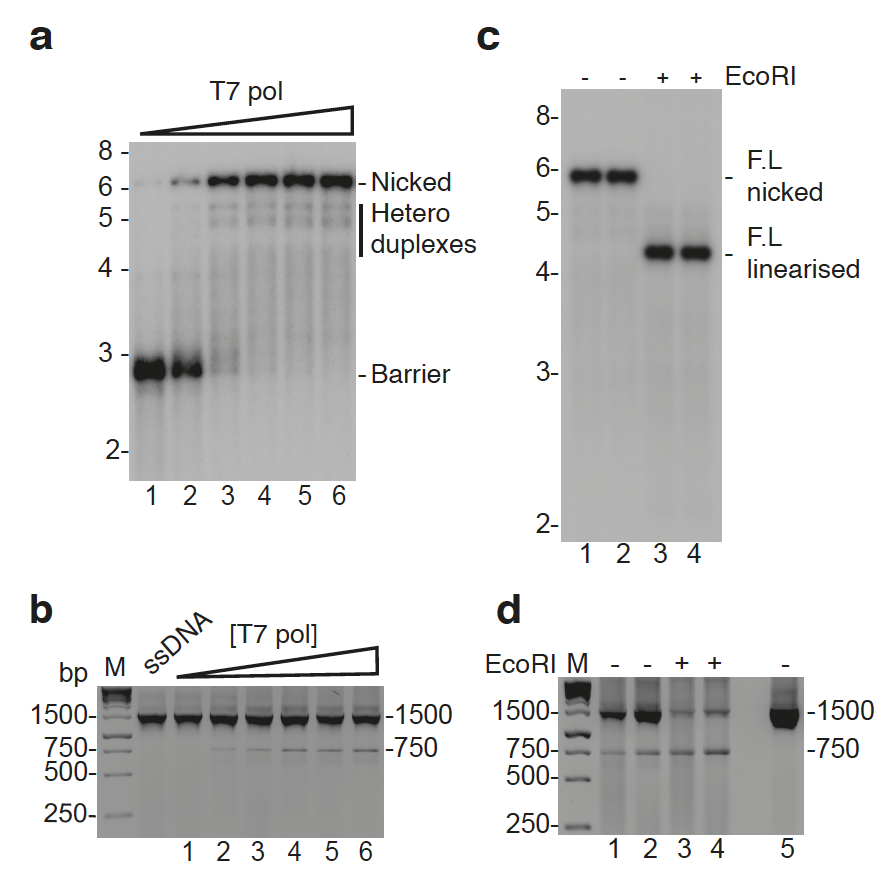
**

**Supplementary Figure 7. Deletion formation using T7 DNA polymerase.**

(a) L-strand DNA synthesis was performed as described in Methods but in the presence of increasing amounts of T7 DNA polymerase (0.015, 0.05, 0.15, 0.25, 0.35, 0.5). (b) The reactions in panel a were analysed by PCR. The T1 substrate template without added T7 DNA polymerase was used as a control. (c) EcoRI cleavage of *in vitro* replication products. L-strand synthesis was performed as described in Fig. 2e, but with T7 DNA polymerase. (d) The reactions in panel c were analysed by PCR before (lane 1 and 2, duplicates) and after EcoRI cleavage (lane 4 and 5, duplicates). Source data are provided as a Source Data file.

**
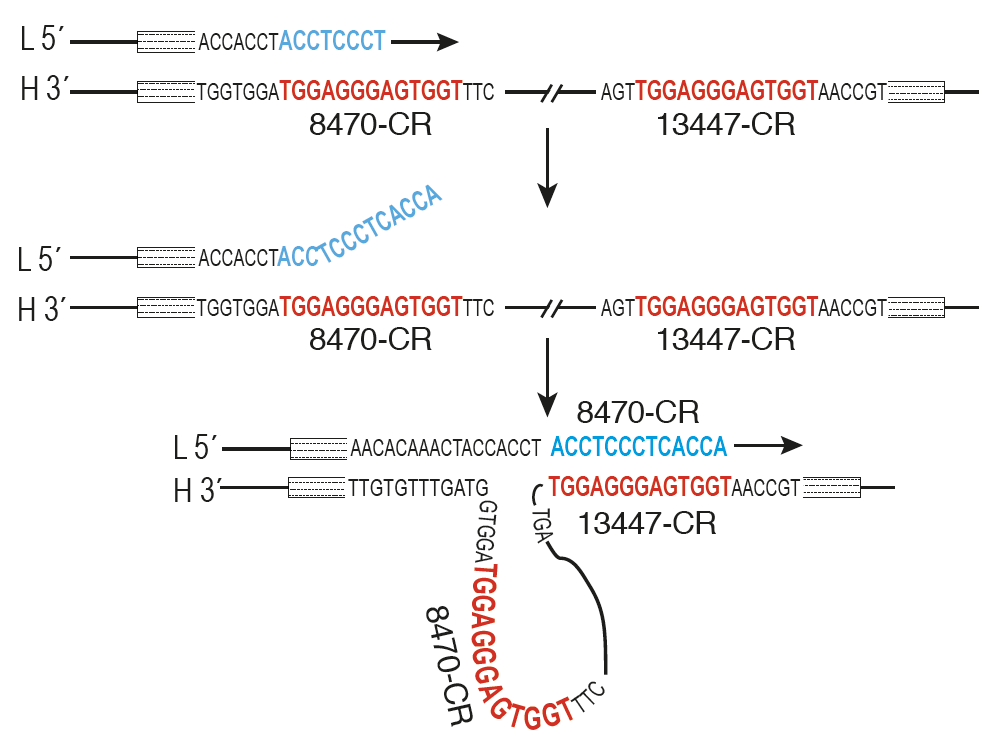
**

**Supplementary Figure 8. Common deletion formation during L-strand synthesis.**

A detailed representation of how the 3′-end of the newly synthesised L-strand can unpair from the first common repeat and realign with the second repeat.

**
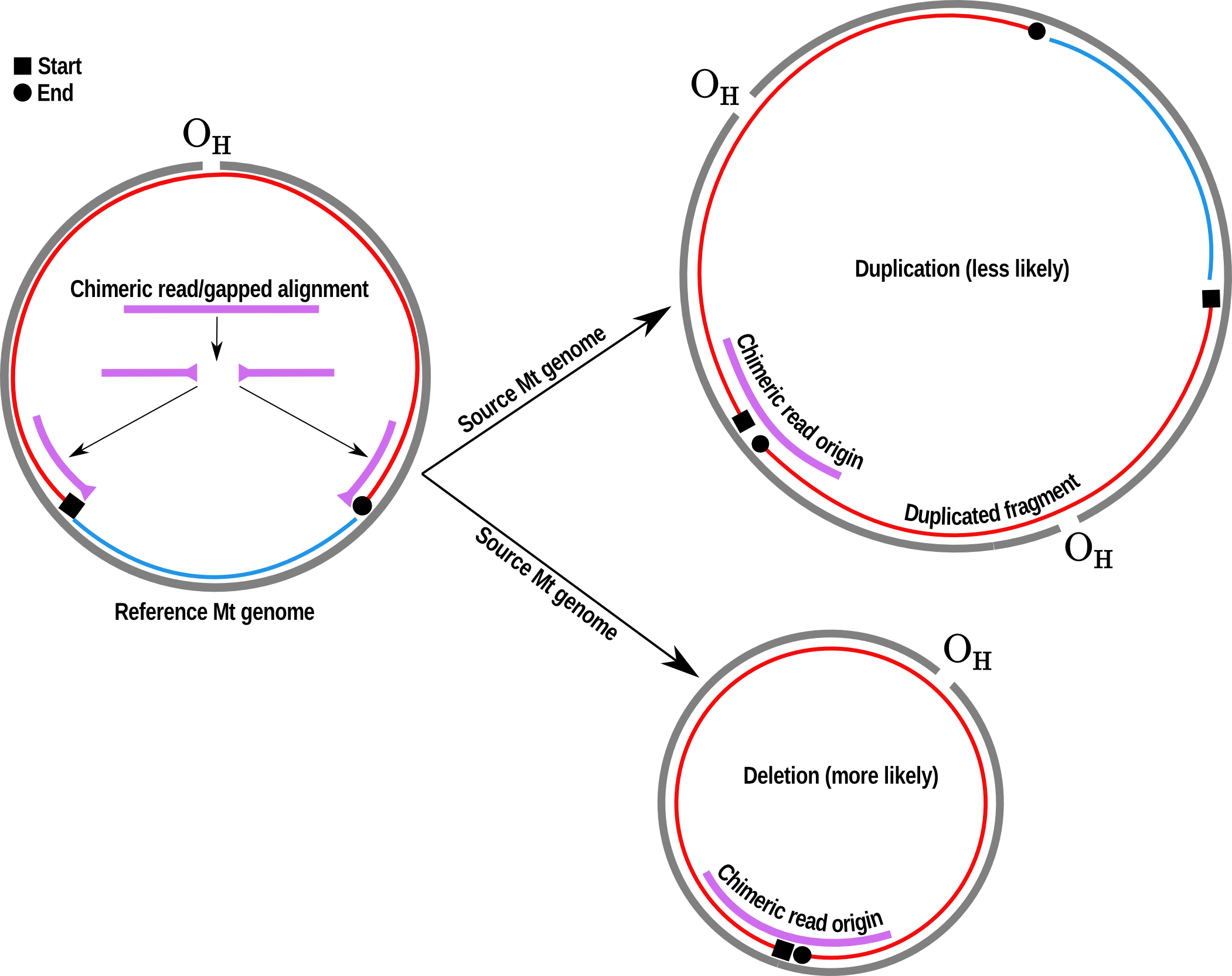
**

**Supplementary Figure. 9. Model used to predict deletions or duplications.**

Mitochondrial genome from whole genome sequencing data was analysed. A gapped alignment of a chimeric read can indicate a deletion of a specific mtDNA arc or alternatively a duplication of the remaining complementary arc. The pipeline initially considers each gapped alignment to represent a deletion, but deletions that lead to removal of origins of replication are re-classified as being likely duplications.

|  | | | |  |
| --- | --- | --- | --- | --- |
| Name | **Derived from** | **Description** |  |  |
| T1, wild type-barrier | pBluescript, mtDNA, IR | Site-directed PCR mutagenesis was used to introduce a GC rich, inverted repeat (IR) sequence of 28 bp (5′-CGC CGC CCA CCC AAC GTA TAC TGG CAA A) next to the common repeats in the T5 WT template, |  |  |
| T2, 8470-CR mut-barrier | pBluescript, mtDNA, IR | Site-directed PCR mutagenesis was used to mutate the 8470-CR sequence to 5′-AAT TAT CTC AAT A. |  |  |
| T3, 13447-CR mut-barrier | pBluescript, mtDNA, IR | Site-directed PCR mutagenesis was used to mutate the 13447-CR sequence to 5′-AAC CTC ATT CAA TA. |  |  |
| T4, 8470-CR and 13447-CR mut-barrier | pBluescript, mtDNA, IR | Site-directed PCR mutagenesis was used to mutate the 8470-CR sequence to 5′-AAT TAT CTC AAT A and the 13447-CR to 5′-AAC CTC ATT CAA TA. |  |  |
| T5, Wild type | pBluescript, mtDNA | MtDNA, position 8184-8788 was cloned between NotI and BamHI, and mtDNA, position 13067-13733 was cloned between ClaI and XhoI in pBluescript SK II-. The two 13 bp common repeats (CR, 5′-ACC TCC CTC ACC A) begin at positions 8470 and 13447. |  |  |
| T6, 8470-CR mut | pBluescript, mtDNA | Site-directed PCR mutagenesis was used to mutate the 8470-CR sequence to 5′-AAT TAT CTC AAT A. |  |  |
| T7, 13447-CR mut | pBluescript, mtDNA | Site-directed PCR mutagenesis was used to mutate the 13447-CR sequence to 5′-AAC CTC ATT CAA TA. |  |  |

**Supplementary Table 1.** Description of templates used for replication experiments. Please note, the sequences in this table follow the revised Cambridge mtDNA reference sequence (NC_012920.1)

Breakpoints Repeat 5′ Sequence 3′ Sequence Deletion class Frequency Ref

T1 template - wild type (750 bp products)

8482-13460 5’ACCTCCCTCACCA ACCTACCTCCCTCACCA[AAG GCGACCTCCCTCACCA]TTGG I (n=164) 43 1

B-(13454) 5’CCC CACGCCGCCC[ACCCAACGT GCGACCTCCC]TCACCATTGG I (n=164) 105 1

B-(13459) 5’CACC CACGCCGCCCACC[CAACGT GCGACCTCCCTCACC]ATTGG I (n=164) 12 1

B-(13454) 5’CCC CACGCCGCCCACCC[AACGT GCGACCTCCC]TCACCATTGG I (n=164) 4 1

T1 template - A467T/T251I+P587L (750 bp products)

8482-13460 5’ACCTCCCTCACCA ACCTACCTCCCTCACCA[AAG GCGACCTCCCTCACCA]TTGG I (n=63) 40 1

B-(13454) 5’CCC CACGCCGCCC[ACCCAACGT GCGACCTCCC]TCACCATTGG I (n=63) 15 1

B-(13459) 5’CACC CACGCCGCCCACC[CAACGT GCGACCTCCCTCACC]ATTGG I (n=63) 6 1

B-(13454) 5’CCC CACGCCGCCCACCC[AACGT GCGACCTCCC]TCACCATTGG I (n=63) 2 1

T1 template – wild type (350 bp products)

T-13557 No repeat CCACCGCGGTGG[CGGCCGC ACAAACGCCTG]AGCCCTATCTA III/II (n=30) 2 12

8234-13631 5’CCCTAA ATTAATTCCCCTAA[AAATCTT AATAATTCTTCTCACCCTAA]CAG I/II (n=30) 21 2,3,4,5

8231-13651 5’TTCCCC ATTAATTCCCC[TAAAAATCTT AACCTCGCTTCCCC]ACCCTTAC I (n=30) 3 5,6,7

8264-13655 5’ACCC CGTATTTACCC[TATAGCACC AACCTCGCTTCCCCACCC]TTAC I (n=30) 1 8,9

8284-13681 5’ACCCC CACCCCCTCTACCCC[CTCTA ACGAAAATAACCCC]ACCCTACT I (n=30) 2 3

8295-13695 5’CCC CTCTAGAGCCC[ACTGTAAAG ACCCTACTAAACCC]CATTAAAC I (n=30) 1 7,10

T5 template – wild type (750 bp products)

8482-13460 5’ACCTCCCTCACCA ACCTACCTCCCTCACCA[AAG AACCTCCCTCACCA]TTGGCAG I (n=82) 74 1

8482-13460 5’ACCTCCCTCACCA ACCAACCTCCCTCACCA[AAG AACCTCCCTCACCA]TTGGCAG I (n=82) 2 1

8482-13460 5’ACCTCCCTCACCA ACCCACCTCCCTCACCA[AAG AACCTCCCTCACCA]TTGGCAG I (n=82) 1 1

8482-13460 5’ACCTCCCTCACCA ACCCACCCCCCTCACCA[AAG AACCTCCCTCACCA]TTGGCAG I (n=82) 1 1

8482-13460 5’ACCTCCCTCACCA ACCCACCTCACTCACCA[AAG AACCTCCCTCACCA]TTGGCAG I (n=82) 1 1

8482-13460 5’ACCTCCCTCACCA ACCTACCTCCCTCACCA[AAG AACCTCCCTCACCA]TCGGCAG I (n=82) 1 1

8398-13684 5’CCCACC CCGTATGGCCCACC[ATAATT AAAATAACCCCACC]CTACTAAA I (n=82)1 3

8283- plasmid 5’TACCC CCCCCTCTACCC[CCTCTAGA GGGCCCGGTACCC]AATTCGCC I (n=82)1 6

T1 template - T7 DNA polymerase (750 bp products)

8482-13460 5’ACCTCCCTCACCA ACCTACCTCCCTCACCA[AAG ACCTCCCTCACCA]TTGGCAG I (n=93) 77 1

B-(13454) 5’CCC CACGCCGCCC[ACCCAACGT ACCTCCC]TCACCATTGGCAG I (n=93) 5 1

B-(13459) 5’CACC CACGCCGCCCACC[CAACGT ACCTCCCTCACC]ATTGGCAG I (n=93) 2 1

B-(13460) 5’CCA CACGCCGCCCACCCA[ACGT ACCTCCCTCACCA]TTGGCAG I (n=93) 9 1

**Supplementary Table 2. Deletions formed *in vitro* detected after PCR, cloning and sanger sequencing.**

All the identified mtDNA repeats involved in breakpoint formation have also been identified in naturally occurring breakpoints *in vivo*. See the mitochondrial DNA breakpoints database for references and details (<http://mitobreak.portugene.com/cgi-bin/Mitobreak_home.cgi>). Repeat sequences are shown in blue. Point mutations are shown in red. Intervening sequences lost during copy-choice recombination are shown in brackets. The frequency of each deletion is indicated. Breakpoints labelled with B indicate that the 5′ repeat is located in the artificial barrier and T that the 5′ repeat is located in the plasmid backbone. The common repeat is underlined.

**REFERENCES**

1. Damas, J., et al. Mitochondrial DNA rearrangements in health and disease--a comprehensive study. *Hum Mutation.* Jan;35 (1):1-14 (2014).

2. Kraytsberg, Y., et al., [Mitochondrial DNA deletions are abundant and cause functional impairment in aged human substantia nigra neurons.](http://www.ncbi.nlm.nih.gov/pubmed/16604072) *Nature Genetics.*38 (5), 518-20 (2006).

3. Moslemi, A.R., C. Lindberg, and A. Oldfors, [Analysis of multiple mitochondrial DNA deletions in inclusion body myositis.](http://www.ncbi.nlm.nih.gov/pubmed/9375854) *Human Mutation.* 10(5), 381-6 (1997)

4. Samuels, D.C., E.A. Schon, and P.F. Chinnery, [Two direct repeats cause most human mtDNA deletions.](http://www.ncbi.nlm.nih.gov/pubmed/15313545) *Trends in Genetics*. 20(9), 393-8 (2004).

5. Reeve, A.K., et al., [Nature of mitochondrial DNA deletions in substantia nigra neurons.](http://www.ncbi.nlm.nih.gov/pubmed/18179904) *American Journal of Human Genetics.* 82(1), 228-35 (2008).

6. Rogounovitch, T.I., et al., [Large deletions in mitochondrial DNA in radiation-associated human thyroid tumors.](http://www.ncbi.nlm.nih.gov/pubmed/12460924) *Cancer Research*. 62(23), 7031-41 (2002).

7. Moslemi, A.R., et al., [Clonal expansion of mitochondrial DNA with multiple deletions in autosomal dominant progressive external ophthalmoplegia.](http://www.ncbi.nlm.nih.gov/pubmed/8957011) *Annals of Neurology.* 40, 707-13 (1996).

8. Fukushima, K. and C. Fiocchi, [Paradoxical decrease of mitochondrial DNA deletions in epithelial cells of active ulcerative colitis patients.](http://www.ncbi.nlm.nih.gov/pubmed/15068964) *American Journal of Physiology - Gastrointestinal and Liver Physiology*. 286(5), G804-13 (2004).

9. Penta, J.S., et al., [Mitochondrial DNA in human malignancy.](http://www.ncbi.nlm.nih.gov/pubmed/11344040) Mutation Research. 488(2), 119-33 (2001).

10. Wanrooij, S., et al.,  [Twinkle and POLG defects enhance age-dependent accumulation of mutations in the control region of mtDNA.](http://www.ncbi.nlm.nih.gov/pubmed/15181170) *Nucleic Acids Research*. 32(10), 3053-64 (2004).
