# Supplementary material for: Copy-choice recombination during mitochondrial L-strand synthesis causes DNA deletions
Source: Nat Commun. 2019 Feb 15;10:759. doi: 10.1038/s41467-019-08673-5 (PMC6377680; doi:10.1038/s41467-019-08673-5)
Supplement: Supplementary file 2 — Description of Additional Supplementary Files [file 41467_2019_8673_MOESM2_ESM.docx]

**Description of Additional Supplementary Files**

File Name: Supplementary Data 1

Description: List of breakpoints identified *in vivo*.

File Name: Supplementary Data 2

Description: List of breakpoints identified *in vitro*.
