# Supplementary material for: Copy-choice recombination during mitochondrial L-strand synthesis causes DNA deletions
Source: Nat Commun. 2019 Feb 15;10:759. doi: 10.1038/s41467-019-08673-5 (PMC6377680; doi:10.1038/s41467-019-08673-5)
Supplement: Supplementary file 6 — Reporting Summary [file 41467_2019_8673_MOESM6_ESM.pdf]

nature research

Corresponding author(s): Maria Falkenberg

Last updated by author(s): Dec 16, 2018

Reporting Summary

Nature Research wishes to improve the reproducibility of the work that we publish. This form provides structure for consistency and transparency in reporting. For further information on Nature Research policies, see Authors & Referees and the Editorial Policy Checklist.

Statistics

For all statistical analyses, confirm that the following items are present in the figure legend, table legend, main text, or Methods section.

n/a	Confirmed	
☐	☒	The exact sample size (*n*) for each experimental group/condition, given as a discrete number and unit of measurement
☐	☒	A statement on whether measurements were taken from distinct samples or whether the same sample was measured repeatedly
☐	☒	The statistical test(s) used AND whether they are one- or two-sided<br>*Only common tests should be described solely by name; describe more complex techniques in the Methods section.*
☒	☐	A description of all covariates tested
☐	☒	A description of any assumptions or corrections, such as tests of normality and adjustment for multiple comparisons
☒	☐	A full description of the statistical parameters including central tendency (e.g. means) or other basic estimates (e.g. regression coefficient) AND variation (e.g. standard deviation) or associated estimates of uncertainty (e.g. confidence intervals)
☒	☐	For null hypothesis testing, the test statistic (e.g. *F*, *t*, *r*) with confidence intervals, effect sizes, degrees of freedom and *P* value noted<br>*Give P values as exact values whenever suitable.*
☒	☐	For Bayesian analysis, information on the choice of priors and Markov chain Monte Carlo settings
☒	☐	For hierarchical and complex designs, identification of the appropriate level for tests and full reporting of outcomes
☒	☐	Estimates of effect sizes (e.g. Cohen's *d*, Pearson's *r*), indicating how they were calculated

Our web collection on statistics for biologists contains articles on many of the points above.

Software and code

Policy information about availability of computer code

Data collection	Data sources are described in Methods
Data analysis	Algorithms and Software used are described in Methods

For manuscripts utilizing custom algorithms or software that are central to the research but not yet described in published literature, software must be made available to editors/reviewers. We strongly encourage code deposition in a community repository (e.g. GitHub). See the Nature Research guidelines for submitting code & software for further information.

Data

Policy information about availability of data

All manuscripts must include a data availability statement. This statement should provide the following information, where applicable:

- Accession codes, unique identifiers, or web links for publicly available datasets
- A list of figures that have associated raw data
- A description of any restrictions on data availability

Data supporting the findings of this study are included in the Source Data File; the Supplementary Data 1 and 2; and in public repositories with accession numbers listed in the Method section.

Field-specific reporting

Please select the one below that is the best fit for your research. If you are not sure, read the appropriate sections before making your selection.

☒ Life sciences ☐ Behavioural & social sciences ☐ Ecological, evolutionary & environmental sciences

For a reference copy of the document with all sections, see nature.com/documents/nr-reporting-summary-flat.pdf

Life sciences study design

All studies must disclose on these points even when the disclosure is negative.

Sample size	Muscle tissue from seven human individuals was used in this study.
Data exclusions	N/A
Replication	All reported in vitro experiments were repeated at least three times.
Randomization	Observations regarding repeat lengths at breakpoints and preferential 5' or 3' localization of breakpoints were assessed relative to datasets with randomly generated breakpoints. 100 random datasets were generated, each having the same number of breakpoints as the observed data.
Blinding	N/A

Reporting for specific materials, systems and methods

We require information from authors about some types of materials, experimental systems and methods used in many studies. Here, indicate whether each material, system or method listed is relevant to your study. If you are not sure if a list item applies to your research, read the appropriate section before selecting a response.

Materials & experimental systems

n/a	Involved in the study	
☒	☐	Antibodies
☒	☐	Eukaryotic cell lines
☒	☐	Palaeontology
☒	☐	Animals and other organisms
☐	☒	Human research participants
☒	☐	Clinical data

Methods

n/a	Involved in the study	
☒	☐	ChIP-seq
☒	☐	Flow cytometry
☒	☐	MRI-based neuroimaging

Human research participants

Policy information about studies involving human research participants

Population characteristics	N/A
Recruitment	These are patients diagnosted by the Newcastle Highly Specialised Mitochondrial Diagnostic laboratory. Alongside clinical colleagues within the Newcastle upon Tyne Hospitals NHS Foundation Trust, the laboratory have been providing a comprehensive, diagnostic and clinical management service for patients with suspected Mitochondrial Disease for nearly 20 years and have been designated as one of three component groups of the Highly Specialised Rare Mitochondrial Disorders of Adults and Children Service since 2007.
Ethics oversight	Informed consent for diagnostic and research studies was obtained for all subjects in accordance with the Declaration of Helsinki protocols and approved by the local institutional review board (Newcastle and North Tyneside Local Research Ethics Committees (reference 2002/205)).

Note that full information on the approval of the study protocol must also be provided in the manuscript.
